# Retrieval of 30 Lymph Nodes Is Mandatory for Selected Stage II Gastric Cancer Patients

**DOI:** 10.3389/fonc.2021.593470

**Published:** 2021-04-30

**Authors:** Yong-He Chen, Jun Lu, Run-Cong Nie, Dan Liu, Ai-Hong Liu, Zi-Jian Deng, Xi-Jie Chen, Jun Xiang, Ying-Bo Chen, Chang-Ming Huang, Shi Chen, Jun-Sheng Peng

**Affiliations:** ^1^ Department of Gastrointestinal Surgery, The Sixth Affiliated Hospital, Sun Yat-Sen University, Guangzhou, China; ^2^ Department of Surgery, Guangdong Institute of Gastroenterology, Guangzhou, China; ^3^ Department of Surgery, Guangdong Provincial Key Laboratory of Colorectal and Pelvic Floor Diseases, Guangzhou, China; ^4^ Department of Gastric Surgery, Fujian Medical University Union Hospital, Fuzhou, China; ^5^ Department of Gastric Surgery, Sun Yat-sen University Cancer Center, State Key Laboratory of Oncology in South China, Collaborative Innovation Center for Cancer Medicine, Guangzhou, China; ^6^ Department of Laboratory Science, The Second Affiliated Hospital, Guangzhou University of Chinese Medicine, Guangzhou, China

**Keywords:** stage II, gastric cancer, prognosis, Chinese people, lymph node retrieval

## Abstract

**Background:**

According to the 8th edition AJCC staging manual, a least of 16 lymph nodes retrieval (LNRs) is the minimal requirement for lymph nodes (LNs) dissection of gastric cancer surgery. Previous studies have shown that increasing the number of LNRs (≥30) prolongs survival for selected patients. However, the necessity of retrieving 30 or more LN for stage II gastric cancer patients is still under debate.

**Aim:**

This study aims to explore the impact of retrieving 30 or more lymph nodes on the survival of stage II cancer patients.

**Methods:**

A total of 1,177 patients diagnosed with stage II gastric cancer were enrolled in this study. The clinicopathological parameters and the impact of different LNRs (<30 or ≥30) and positive lymph node ratio (NR) on overall survival (OS) were retrospectively analyzed.

**Results:**

The mean number of LNRs was 34 ± 15. A total of 44% (518/1,177) of patients had an LNRs <30, while 56% (659/1,177) of patients had an LNRs ≥30. The 5-year survival rate was 81% for all patients, 76% for the LNRs <30 group, and 86% for LNRs ≥30 group, respectively (P = 0.003). The survival benefit of retrieving 30 lymph nodes was significant in certain subgroups: age >60 years/male/underwent total gastrectomy/stage IIB. For N+ patients, higher NR was significantly correlated with poor survival.

**Conclusion:**

The survival benefit of retrieving 30 LNs varies in different subgroups. An LNRs of 30 is mandatory for selected stage II gastric cancer patients.

## Introduction

Gastric cancer is the fourth most common malignant tumor in the world and is one of the most common causes of cancer-related death (1). Lymphatic metastasis is a major metastatic pathway for gastric cancer. Extended lymph node dissection is an important part of radical gastrectomy. According to the 8^th^ edition of the AJCC staging manual, the retrieval of at least 16 lymph nodes is the minimal requirement for lymph node dissection, and retrieval of 30 lymph nodes is more desirable (2). Studies have found that an increase in the number of lymph nodes retrieved is associated with prolonged survival (3–10) because more lymph nodes retrieved may indicate more extended lymph node dissection and may help avoid tumor migration to a lower stage than the actual stage. Thus, some studies have proposed an argument that the minimal number of LNRs should be improved to a higher standard. However, there are also some studies stating that an increase in the LNRs only benefits certain groups of patients. Macalindong et al. found in a high-volume gastric cancer data set that retrieving 30 or more LNs did not influence the survival of stage II GC patients (4). Vuong et al. also found that retrieving 30 or more LNs only benefited those with a more advanced N stage (11). The attempt to harvest more LNs may bring forth more postoperative complications (6, 12). To date, the necessity of retrieving 30 or more LNs for stage II gastric cancer patients is still debatable.

In our opinion, the minimal requirement for lymph node retrieval should be individualized according to the characteristics of the patients and the tumor.

This study aims to explore the impact of retrieving 30 or more lymph nodes on the survival of selected stage II cancer patients.

## Materials and Methods

### Study Population and Data Collection

A total of 1,177 patients who received curative surgery from April 2008 to May 2017 were identified from the gastrointestinal malignancy cancer database of The Sixth Affiliated Hospital of Sun Yat-Sen University, Fujian Medical University Union Hospital, and Sun Yat-Sen University Cancer Center.

The inclusion criteria were as follows: (i) patients with histologically confirmed adenocarcinoma of the stomach or esophagogastric junction; (ii) patients who underwent gastrectomy with standardized D2 lymphadenectomy; and (iii) a post-surgery pathological stage of II according to the 8^th^ edition of the AJCC staging manual. The exclusion criteria were as follows: (i) patients with insufficient information; (ii) patients who received preoperative neoadjuvant therapy.

After initial screening, a total of 1,177 patients were included in this study. All available clinical information was retrieved from the database, including general patient demographics, tumor location, differentiation, tumor size, resection extent, LNRs, tumor stage, and survival. The patient information is listed in **Table 1**. The primary endpoint of this study is overall survival. Post-operative Surveillance followed the recommendation of the National Comprehensive Cancer Network guideline (13). Follow-up visits for all three institutions generally consist of clinic visits every 6 months for the first 2 years and annually up to 5 years. Most routine patient follow-up appointments include a physical examination, laboratory tests, chest-abdominal computed tomography scan, and an annual endoscopic examination. In all the three institutions involved in this study, patients’ follow-up was conducted by the staff of the follow-up offices. After the surgery, the follow-up office generally contacts the patients or the patients’ families every 6 months, by telephone calls or mails, to gather information on the patients’ condition and survival. This retrospective study was approved by the Institutional Review Board of all three centers.

**Table 1:** Patient characteristics and P value of univariate analysis.

Subgroups	Lymph nodes retrieved <30 (n = 518)	Lymph nodes retrieved ≥30 (n = 659)	Total (n = 1,177)	P-value
Sex (%)				
* Male*	377 (72.8)	474 (71.9)	851 (72.3)	0.796
* Female*	141 (27.2)	185 (28.1)	326 (27.7)	
Age (years)	60.7 ± 11.8	58.6 ± 11.5	59.5 ± 11.7	0.002
Tumor location (%)				
* Upper third*	178 (34.4)	184 (27.9)	362 (30.8)	0.007
* Middle third*	63 (12.2)	123 (18.7)	186 (15.8)	
* Lower third*	251 (48.5)	314 (47.6)	565 (48.0)	
* Whole stomach*	26 (5.0)	38 (5.8)	64 (5.4)	
Tumor size(cm)	4.26 ± 2.22	4.41 ± 2.15	4.34 ± 2.18	0.22
Tumor differentiation (%)				
* Poorly*	255 (49.2)	344 (52.2)	599 (50.9)	0.158
* Moderately*	231 (44.6)	291 (44.2)	522 (44.4)	
* Well*	5 (1.0)	6 (0.9)	11 (0.9)	
* Data missing*	27 (5.2)	18 (2.7)	45 (3.8)	
Extend of gastrectomy (%)				
* Distal*	271 (52.3)	338 (51.3)	609 (51.7)	0.001
* Proximal*	65 (12.5)	42 (6.4)	107 (9.1)	
* Total*	177 (34.2)	274 (41.6)	451 (38.3)	
* Data missing*	5 (1.0)	5 (0.8)	10 (0.8)	
Adjuvant chemotherapy (%)				
* Yes*	372 (71.8)	456 (69.2)	828 (70.3)	0.336
* No*	146 (28.2)	203 (30.8)	349 (29.7)	
Lymph node retrieved	21 ± 6	44 ± 12	34 ± 15	<0.001
Positive lymph node	1.1 ± 0.08	1.5 ± 0.09	1.3 ± 0.06	0.007
NR	5.7 ± 0.4	3.7 ± 0.2	4.60 ± 0.2	<0.001
Stage IIA (%)				
* pT1N2M0*	27 (5.2)	47 (7.1)	74 (6.3)	0.206
* pT2N1M0*	64 (12.4)	73 (11.1)	137 (11.6)	0.546
* pT3N0M0*	155 (29.9)	193 (29.3)	348 (29.6)	0.861
Stage IIB (%)				
* pT1N3M0*	7 (1.4)	22 (3.3)	29 (2.5)	0.03
* pT2N2M0*	33 (6.4)	52 (7.9)	85 (7.2)	0.352
* pT3N1M0*	113 (21.8)	166 (25.2)	279 (23.7)	0.288
* pT4aN0M0*	119 (23.0)	106 (16.1)	225 (19.1)	0.01

### Surgery

All patients received total or subtotal gastrectomy with D2 lymphadenectomy following the guidelines of the Japanese Gastric Cancer Association (14) (open or laparoscopic surgery depending on the surgeon’s preference). A thorough examination of the abdominal cavity was routinely performed to determine the status of peritoneal metastasis. Peritoneal washing cytology tests were not routinely conducted. The extent of gastric resection was determined by the anatomical location of the tumor. Proximal or total gastrectomy with esophagogastrostomy or Roux-en-Y esophagojejunostomy reconstruction was performed for tumors located in the upper or middle third part of the stomach; distal subtotal gastrectomy with Billroth I, Billroth II, or Roux-en-Y gastrojejunostomy reconstruction was performed for tumors located in the distal third part of the stomach.

### Specimens Assessment

Retrieval of lymph nodes from the gross specimens was by manual method, which means operators identified suspicious lymph nodes by sight and palpation and reconfirmed them under microscopic view. Station labeling was determined according to the anatomical sites and their relationship to the perigastric vessels. This procedure was performed by the surgeons on the fresh specimens instantly after surgical resection. Lymph nodes removed individually during surgery are labeled for stations by the surgeons and inspected separately. Pathological staging was determined according to the AJCC TNM staging system (2). In the final analysis, the patients were divided into two subgroups: patients with less than 30 lymph nodes retrieved (LNRs <30 group) and patients with 30 or more lymph nodes retrieved (LNRs ≥30 group).

### Data Analysis

The clinicopathological characteristics were compared using χ2 tests for categorical variables and analysis of variance (ANOVA) for continuous variables, as appropriate. Patients were divided into different subgroups according to the clinicopathological features (age, sex, the extent of gastrectomy, tumor location, tumor differentiation, T stage, and N stage). For each subgroup, the Kaplan-Meier method and log-rank test were used to assess the survival impact of different lymph node retrievals (LNRs <30 *versus* LNRs ≥30). The hazard ratio (HR) and the 95% confidence interval (95% CI) were calculated by the Cox proportional hazards model, upon which a forest plot was built to visualize the impact on survival. A P value < 0.05 was considered statistically significant. Statistical analysis was performed with SPSS version 25.0 for Windows (IBM, Armonk, NY, USA).

## Results

### Patient Characteristics

**Figure 1:**
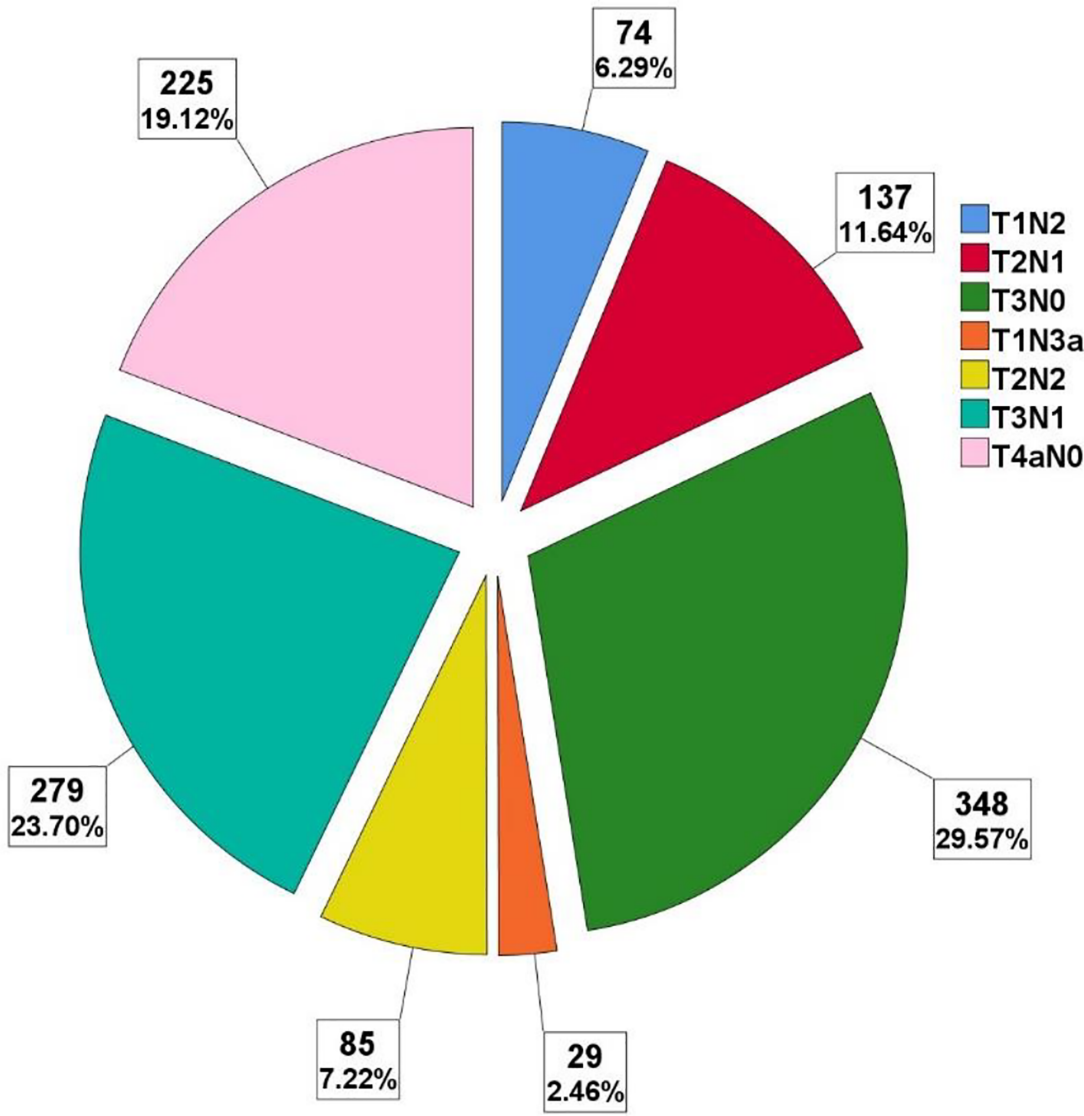
Cases and proportion of different pTN stages.

A total of 1,177 patients were enrolled in this study. The majority of patients were male. The patients’ median age at diagnosis was 59 years (range, 16 to 91 years). Nearly half of the tumors were located in the distal part of the stomach, also half of the tumors were poorly differentiated, other details are depicted in **Table 1**. The spectrum of the specific pTNM stage is shown in **Figure 1**, different pTN stages were roughly balanced in the LNRs ≥30 and <30 subgroups.

### Lymph Node Retrieval

**Figure 2:**
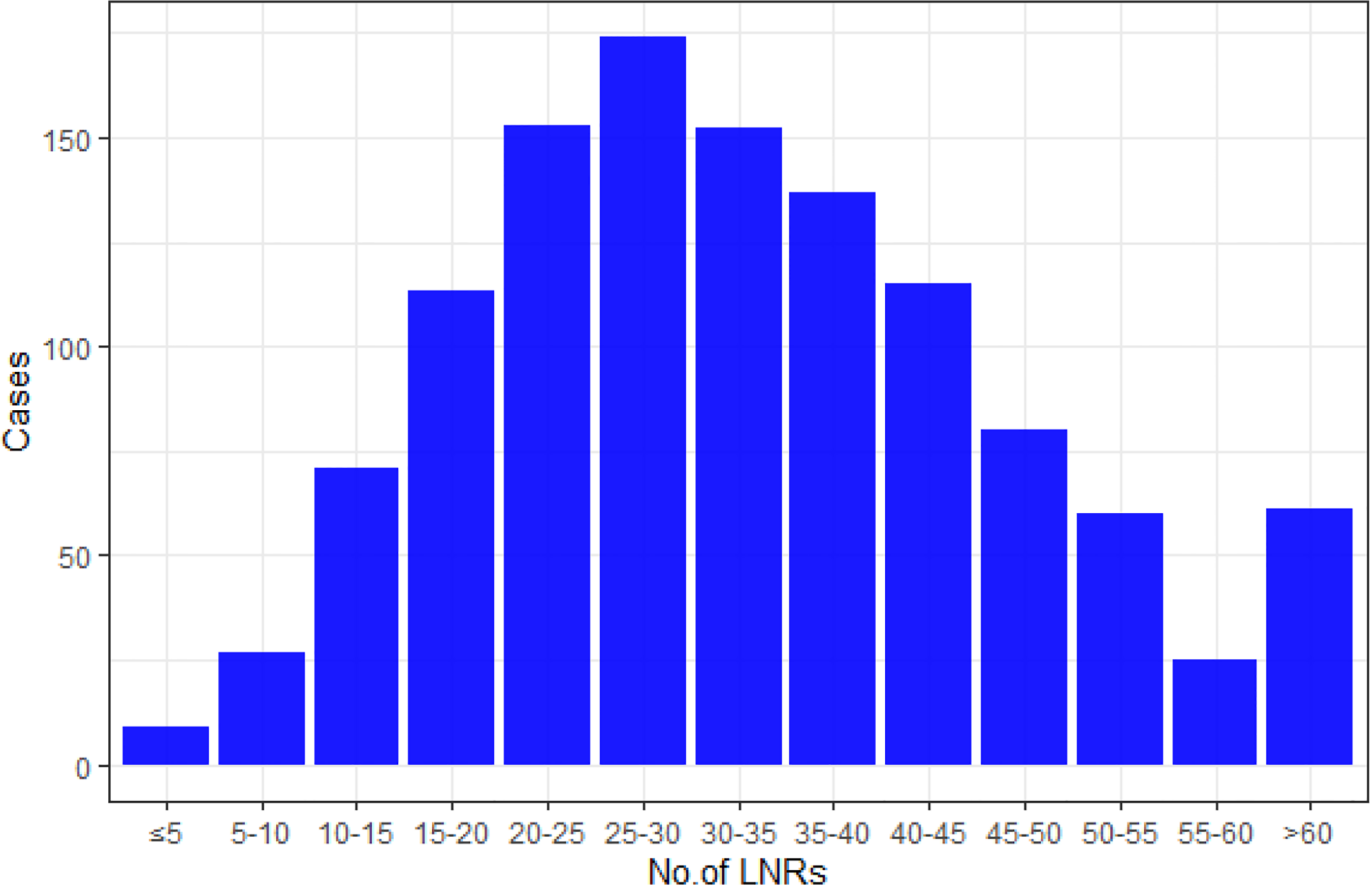
The distribution of patients according to the number of LNRs.

**Figure 3:**
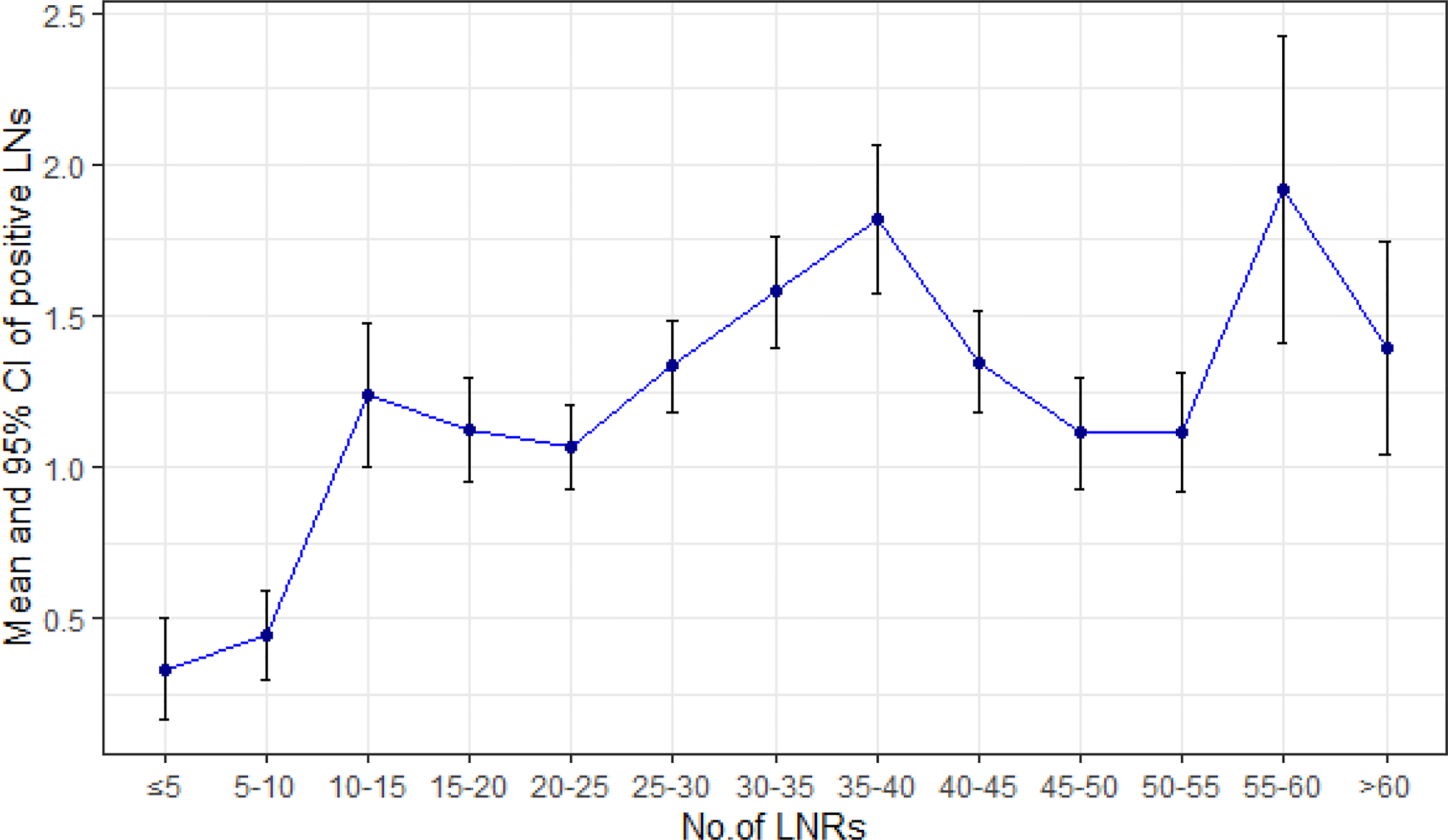
Increasing pattern of mean numbers of positive LNs according to the number of LNRs.

The mean number of LNRs was 34 ± 15, with a range from 3 to 101. Numbers of patients with LNRs <30 (518/1,177, 44%) and LNRs ≥30 (659/1,177, 56%) were roughly balanced. The distribution of LNRs and numbers of positive lymph nodes are shown in **Figures 2**, **3**, respectively. The numbers of positive lymph nodes showed a tendency of increasing as the LNRs increased. In the LNRs ≥30 subgroups, with the increase in the numbers of total LNRs, the numbers of positive lymph nodes were slightly higher, but the positive lymph nodes ratio (NR) was significantly lower than the LNRs <30 subgroup.

### Survival Analyses

**Figure 4:**
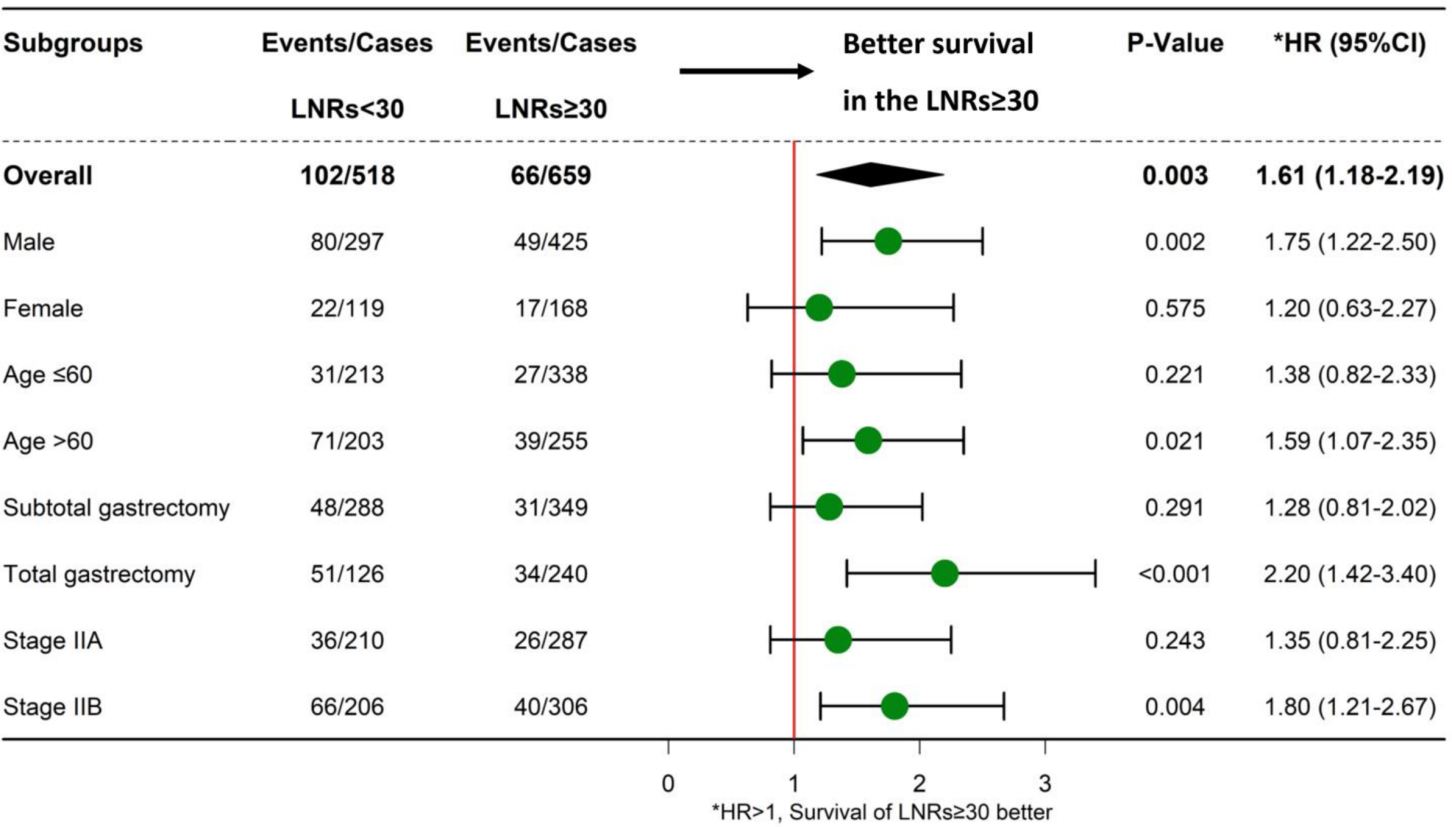
Forest plot showing the impact of LNRs (<30 or LNRs ≥30) on survival in different subgroups.

**Figure 5:**
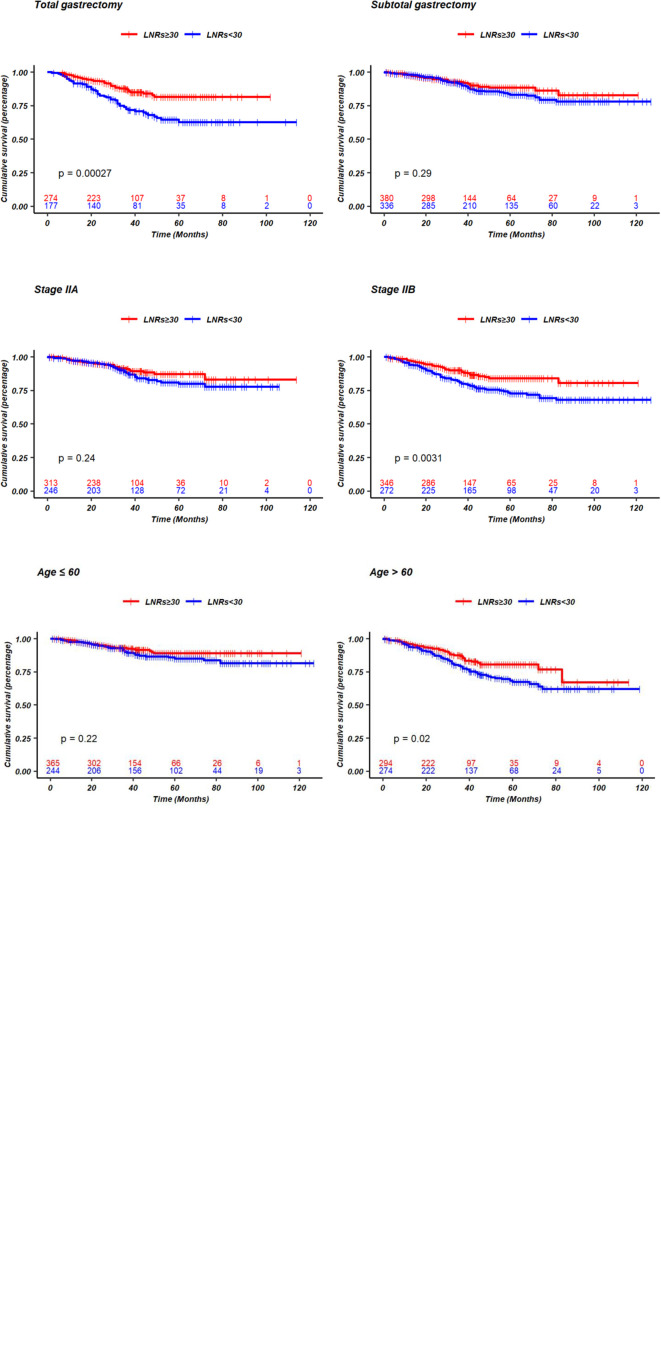
Kaplan-Meier curves of survival comparing LNRs <30 and LNRs ≥30 stratified by sex, age, extend of gastrectomy, different pTN stage.

**Figure 6:**
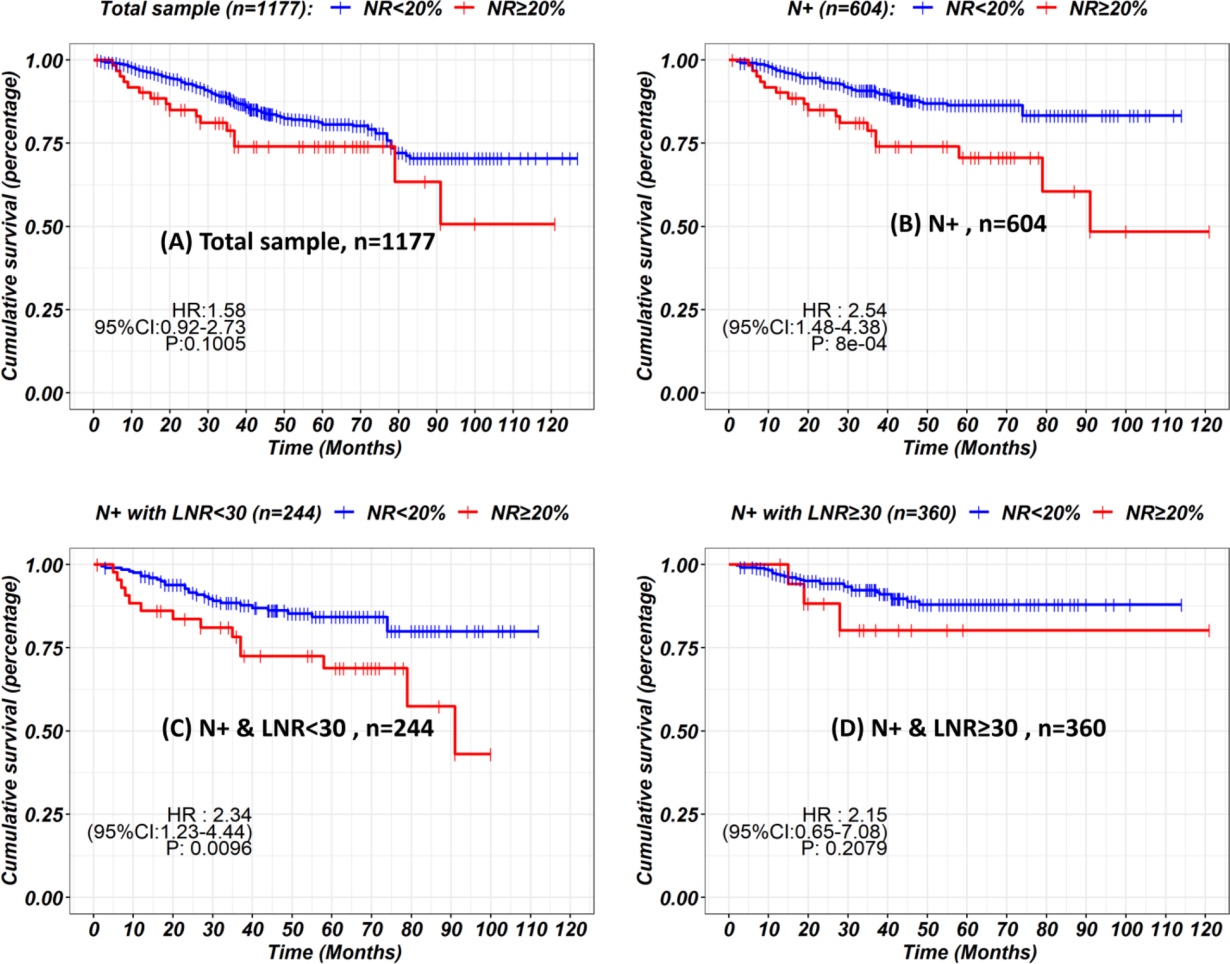
Kaplan-Meier curves showing the impact of node ratio on survival in the total sample **(A)**, N+ subgroups **(B)** stratified by LNRs **(C, D)**.

Up to January 2020, in a median follow-up time of 44 months, a total of 168 tumor-related death events were observed in the 1,177 patients. The 5-year survival rate was 81% for all patients, 76% for the LNRs <30 group, and 86% for LNRs≥30 group, respectively (P = 0.003). The survival and hazard ratios adjusted by subgroups are depicted in **Figures 4**, **5**. Although overall speaking, survival was significantly improved in the LNRs ≥30 group, it was clear that the survival benefit of retrieving 30 LNs varied in different subgroups. Patients aged >60 years/male/who underwent total gastrectomy/stage IIB tended to benefit from an increase in the number of LNRs. The impact of NR on survival is not statistically significant in the total sample, but in the subgroup analysis, NR is significantly correlated with worse survival in the pN+ subgroup, especially for the N+ patients with LNRs <30, as depicted in **Figure 6**.

## Discussion

According to the 8^th^ edition of the AJCC staging manual, retrieval of at least 16 LNs is the minimal requirement after curative resection surgery (14, 15). However, gastric cancer is a malignancy with great heterogeneity, and applying the same standard to all patients in different conditions may lead to treatment bias. Previous studies have shown that increasing the LNRs number is significantly correlated with prolonged survival for patients (3–10). Some researchers believe that retrieval of at least 16 LNs is not sufficient to warrant adequate lymphadenectomy extension and accurate pathological staging. Retrieval of more LNs (>25 or 30) is mandatory, while some researchers argue that retrieving more LNs only benefits those at more advanced stages (stage III), and attempting to retrieve 30 LNs for all patients is unnecessary. For example, Macalindong et al. found that the 5-year disease-free survival rate was not significantly different between the LNRs <30, LNRs 30–45, and LNRs >45 groups (72.9 *vs* 79.2 *vs* 76.2%, P value = 0.566) in stage II patients (4). Vuong et al. also found that retrieving 30 or more LNs only resulted in a benefit for patients with pT1N3 and pT2N3 GC (11). Thus, the benefit of 30 LNRs is still controversial for stage II GC patients.

In this study, we analyzed the survival impact of retrieving 30 or more LNs on the largest stage II gastric cancer cohort ever reported. A total of 88.7% (1,155/1,302) of patients had an LNRs ≥16, and 53.4% (695/1,302) had an LNRs ≥30, which is superior to previous reports, in which only 23–45% of patients had an LNRs >16 (5, 16). Our finding is that retrieving 30 or more LNs is beneficial for stage II gastric cancer patients as a total group.

Increasing the number of LNRs benefits patients by providing a more accurate N stage and more extended lymphadenectomy. If hypothetically, we assume that the N stage is accurate when the number of LNRs is more than 16, then the mean number of positive LNs should remain steady when the LNRs are above 16. However, our data show that the mean number of positive LNs is still increasing when the LNRs are above 16, indicating that the N stage is still not reliable even if a minimum of 16 LNRs is met according to the AJCC staging manual. An insufficient LNRs will lead to stage migration, affecting the expected prognosis, sequential adjuvant therapy, and monitoring. Our data showed that the survival impact of NR was not significant in the total sample, but further subgroup analysis showed that higher NR was correlated with poor survival in pN+ subgroup, especially in the pN+ subgroup with LNRs <30. This could be explained by the constitution of stage II patients. As depicted in our data, approximately half of the stage II patients are with pN0 stage and a NR of 0%, thus, the prognosis predictive value of NR is limited. But for the pN+ patients with insufficient LNRs, introducing an index of positive lymph node ratio (NR) could potentially overcome the bias of inaccurate N staging, which is in concordance with the previous reports (17, 18). Nevertheless, the efforts by the surgeons and pathologists to retrieve more lymph nodes were important for avoiding false pN staging (19). To retrieve more lymph nodes, continuous cooperation between surgeons and pathologists is necessary. Surgeons should make their efforts to retrieve more lymph node during surgery following the standard of D2 regional lymphadenectomy (20). Adopting operative lymphatic tracer such as indocyanine green or carbon nanoparticles during lymphadenectomy had shown a great potential of increasing lymph node retrieval (21, 22). After surgery, it is highly recommended that the surgeons should handle the procedure of separating lymph node stations instantly on the fresh specimen, to improve LNRs and accuracy of station labeling (23–25).

Another important finding of our study is that the survival benefit of LNRs ≥30 varies between different subgroups. For patients who underwent total gastrectomy, lymphadenectomy must be more extended according to the Japanese gastric cancer treatment guidelines (14). Thus, patients who receive total gastrectomy should undergo harvest of more LNRs than those who receive subtotal gastrectomy (6, 26). Bouvier et al. reported that the LNRs in total gastrectomy was higher than that of subtotal gastrectomy (10.4 in total gastrectomy; 7.2 in proximal gastrectomy; 7.4 in distal gastrectomy, P value < 0.0001), which is in line with our findings (34.7 in total gastrectomy; 32.7 in subtotal gastrectomy, P value = 0.024). Patients with stage IIB disease were associated with a more advanced disease stage in the stage II group; thus, a more thorough and extended lymphadenectomy is warranted (27, 28), and an LNRs ≥30 is mandatory. Additionally, an LNRs ≥30 also benefits male/age >60 elderly subgroups of patients.

There are a few limitations to our research. First, owing to the nature of the retrospective study design and the different origins of the data sets, treatment bias was inevitable. Second, the effect of confounding factors could not be eliminated in the subgroup analysis. Third, selection bias was also not neglectable because all patients enrolled were from three high-volume gastric cancer centers, and the high number of LNRs and ideal OS may not be easily reproducible in all centers.

## Conclusion

Retrieval of 30 lymph nodes is mandatory for selected stage II gastric cancer patients.

## Data Availability Statement

The raw data supporting the conclusions of this article will be made available by the authors, without undue reservation.

## Ethics Statement

The studies involving human participants were reviewed and approved by the ethics committee of Sun Yat-Sen University Sixth Affiliated Hospital. The patients/participants provided their written informed consent to participate in this study. Written informed consent was obtained from the individual(s) for the publication of any potentially identifiable images or data included in this article.

## Author Contributions

J-SP and SC designed the study; Y-HC, JL and R-CN acquired the data; Y-HC analyzed, interpreted the data, and drafted the initial manuscript. DL made substantial revisions to the manuscript. A-HL, Z-JD, X-JC, JX, Y-BC, and C-MH helped with interpreting the data. Y-HC, JL, and R-CN contributed equally to this work.

## Funding

Supported by Guangzhou Science and Technology Project (grant number 201803010040) and Nation Key Clinical Discipline.

## Conflict of Interest

The authors declare that the research was conducted in the absence of any commercial or financial relationships that could be construed as a potential conflict of interest.
